# Editorial Commentary: Resource utilization and outcomes of intoxicated drivers: does evidence of alcohol-impaired driving affect road traffic crash injury outcomes?

**DOI:** 10.1186/1752-2897-4-10

**Published:** 2010-08-05

**Authors:** Uli Schmucker

**Affiliations:** 1 University Hospital Greifswald, Dept. Trauma Surgery, Road Traffic Crash Research Unit, Sauerbruchstrasse, Germany

## 

Cherry et al. report the results of a retrospective hospital-based study among a sample of 623 alcohol-impaired drivers versus 364 sober drivers, all of which suffered from road traffic injury and were documented with a comparable mean Injury Severity Score (ISS). As for the key results, the alcohol-impaired drivers were more likely to be admitted to the ICU, but less likely to go to the operating room. In addition, they were registered with less ICU days, ventilator days, and hospital days. On the other hand, the impaired drivers were less likely to utilize follow-up care (e.g. rehabilitation) and generate payments for hospital fees.

The objective of this editorial comment is to assist in putting the controversial reports, many of them cited by Cherry et al. [1], of alcohol as a protective factor into a more comprehensive context according to the following points.

(i) There is evidence that alcohol-impaired driving is a strong predictor for involvement in a road traffic crash and severe injury. (ii) There is evidence that crashes associated with alcohol-impairment are different from other crashes in terms of the drivers' age and sex, crash mechanism, type of vehicles involved, use of restraints, etc. (iii) There is no evidence that crash-biomechanics differ from crashes caused by impaired or sober drivers. (iv) There is reason to believe that injuries from road trauma behave similar to those from other trauma. (v) There is reason to believe that evidence or suspicion of acute alcohol intoxication triggers comprehensive trauma management (e.g. transfer to trauma centre, early endotracheal intubation, ICU admission). (vi) There is evidence that alcohol consumption is associated with lower socio-economic status and presence of co-morbidities.

The two latter points might explain the results reported by Cherry et al., meaning that the intoxicated casualty is more likely to be provided with a temporary/definitive airway irrespective of acute injuries. Although the impact of social detriments on trauma care cannot be derived from the data presented, lower socio-economic status is known to trigger early demission from hospital to home. Last but not least, intoxicated drivers tend to be of younger age compared to other injured drivers in emergency department samples. This is consistent with Cherry's sample and might explain the trend of decreasing mortality in the intoxicated sample. Age, in fact, is not represented in the injury scores used by Cherry et al., although it does evidently affect injury outcomes.

In conclusion, the current evidence does not support either of the proposed impacts of alcohol as a protective or non-protective agent with respect to injury outcomes. Despite the independent effect of alcohol on crash risk, future studies should focus on the alcohol-injury-complex irrespective of injury mechanism. In fact, inclusion criteria based on alcohol-levels lead to strong selection biases since drunk-drivers are predominantly male, young, and healthy. Representative samples and multivariate analyses are required to quantify (differentiate!) the impact of acute intoxication and chronic alcohol disease, age group and gender, co-morbidities, and socioeconomic status (e.g. access to health care, insurance status). The latter is believed to have a major impact, although it has been neglected in many previous investigations. Last but not least, drinking alcohol is evidently associated with other risky attitudes, habits, and behaviors (e.g. smoking), all of which may substantially affect injury outcomes. Large-scale studies that control a broad spectrum of injury-independent variables are needed to understand the biologic impact of substances on the pathophysiology of trauma and outcomes.

## Conflict of interests

The author declares that they have no competing interests.

## References

[B1] Cherry RA Nichols PA Snavely TM Camera LJ Mauger DT Resource Utilization and Outcomes of Intoxicated Drivers Journal of Trauma Management & Outcomes 2010 4 9 10.1186/1752-2897-4-9 PMC2924262 20687912

